# A preliminary, quantitative study on the use of traditional and complementary medicine by cancer patients seen at the Senkatana oncology clinic, Maseru, Lesotho

**DOI:** 10.1186/s12906-024-04388-3

**Published:** 2024-04-01

**Authors:** Mopa A. Sooro, Thabo S. Thoahlane, Maseabata V. Ramathebane, Kabelo A. Mputsoe

**Affiliations:** 1 Faculty of Health Sciences, Department of Pharmacy, National University of Lesotho, Roma, Lesotho; 2 Senkatana Oncology Clinic, Maseru, Lesotho

**Keywords:** Cancer, Complementary and alternative medicine, Traditional medicine, Chemotherapy

## Abstract

**Background:**

The use of traditional and complementary medicine (TCM) by cancer patients remains common in several countries especially in the Sub-Saharan Africa. However, the reasons for use are complex and change with time and geographic location, they may vary from therapy to therapy, and they are different from one individual to another. The use of TCM has been associated with active coping behaviour and a way through which patients take control of their own health. However, cancer patients do not disclose their use of TCM to the attending healthcare professionals and therefore the effects of these medicines on the patients may not be ascertained.

**Aim:**

To investigate the use of traditional and complementary medicines among patients diagnosed with cancer.

**Methods:**

A cross-sectional, quantitative study was conducted at Senkatana Oncology clinic in May to June 2023. Cancer patients underwent standardized, quantitative interviews using structured questionnaires about their use of TCM. Descriptive statistics were used to analyse the data. Logistic regression analysis was also used to identify factors associated with satisfaction with the performance of TCM.

**Results:**

All interviewed patients (*n* = 50, 100%) reported to be using TCM. Patients consisted of 24 females (48%) and 26 males (52%) in the age range 14 to 82 years old. The majority of the study population was in the age group 35–44 years old. The most prevalent cancer among participating males was prostate cancer and among females was cervical cancer. Biological products use was the most prominent with the highest average percentage usage (14.7%). The majority of patients (66%, *n* = 33) indicated that they just wanted to try everything that could help. Patients (*n* = 47, 94%) further reported that they had been using complementary medicine during the same period as they were using conventional treatment so that both may work to help each other. Neither gender nor age predicted satisfaction with the performance of traditional and complementary medicine.

**Conclusions:**

It is concluded that all interviewed cancer patients use TCM. Patients indicated that one of the reasons for using TCM was that they wanted to try everything that could help in their cancer care. Patients further reported that they did not inform their oncologist of their concurrent use of TCM because they had been advised not to use other medicines besides what they are given at the clinic.

**Supplementary Information:**

The online version contains supplementary material available at 10.1186/s12906-024-04388-3.

## Background

Cancer remains the leading cause of death globally although its burden varies amongst different countries of the world [1]. In low- and middle-income countries (LMICs) especially in Sub Saharan Africa (SSA), the incidence of cancer has been lower than that of the High-income countries (HIC) [2]. However, LMICs bear a larger burden of cancer mortality than HICs as 70% of the global cancer deaths occur in LMICs [3]. In SSA, a major increase in cancer mortality is estimated from 520 348 in 2020 to about 1 million deaths per year in 2023, unless rapid interventions are implemented [4]. Based on WHO Global Burden of Disease estimates, cancer accounts for 17.2% of non-communicable diseases (NCDs) and 4% of all deaths in Lesotho [5]. In Lesotho, the most common cancer is cervical cancer, followed by breast cancer and prostate cancer accounting for more than 60% of all cases of cancer in 2020 [6]. In July 2022, Lesotho began treatment of patients with solid tumours like breast cancer at its first cancer treatment centre; Senkatana Oncology Clinic. A project to establish this cancer treatment centre started in June 2020 was funded by the Bristol-Myers Squibb Foundation (BMSF) [7]. Lesotho had been relying heavily on the surrounding country; South Africa for the diagnosis and/or treatment of majority of the patients.

In addition to the innate urge among human being to try new and alternative ways of relieving suffering, the barriers to access local conventional oncology services may lead to a number of cancer patients resorting to traditional and complementary medicine (TCM) [8, 9]. The world health organization (WHO) defines TCM as the sum total of the knowledge, skills, and practices based on the theories, beliefs, and experiences traditional to different cultures [10, 11]. These may be explicable or not, and are used in the maintenance of health as well as in the prevention, diagnosis, improvement or treatment of physical and mental illness [11].Complementary medicine is a broad set of healthcare practices that are not part of that country’s own tradition or conventional medicine and are not fully integrated into the dominant health-care system [10, 11]. In many countries, the standard of cancer care is based on conventional medicine which is a system in which medical doctors and other healthcare professionals (such as nurses, pharmacists and therapists) treat symptoms and diseases using drugs, radiation or surgery [12]. In most African countries TCM is considered more compatible to indigenous values and beliefs towards health and life [9]. Traditional herbs (medicinal plants) and remedies are regarded as harmless, lacking adverse effects to health [8].

Some of the factors that have been reported to facilitate increasing use of TCM among cancer patients include to get rid of cancer symptoms such as pain, to cure cancer, improve physical and psychological well-being, treat toxicity of conventional cancer therapies and improve immunity [8]. Some studies have reported the use of TCM as related to a paradigm to disease causation where by chronic disease including cancer is attributed to poor diets, poor lifestyle practices, heredity, physical factors, the environment, spiritual factors and psychological factors [13, 14]. In most LMICs, traditional and complementary medicine is more readily accessible compared to conventional oncology medicine. In addition to the high cost of conventional therapies, these therapies are also highly regulated by law while TCM is often affordable and unregulated [14]. Furthermore, TCM practitioners often do not document their diagnosis and prescriptions, making their work untraceable. Patients also often do not disclose their use of TCM to the attending healthcare professionals [8, 13, 14]. Recent studies report disclosure of the use of TCM to attending healthcare professionals as low as 32% among cancer patients in SSA [8, 13]. The reasons that patients gave for non-disclosure include that their doctors advise against the use of TCM or that they did not ask them about this and so they found no need to inform them [8].

The use of complementary and alternative medicines had been associated with suboptimal clinical outcome or disease resistance [15]. Shark cartilage had been one of the popular complementary or alternative medicine in cancer, and few decades ago, a clinical trial was conducted to study its efficacy in advanced cancer [16]. There was no difference in overall survival between patients receiving standard care plus a shark cartilage product versus standard care plus placebo [15]. Evidence further suggests that the efficacy of chemotherapeutic and hormonal treatment could be reduced due to some drug-herbal or vitamin interactions and that antioxidants and vitamin C during radiation therapy could negatively impact the outcomes [17]. However, the use of traditional, complementary and alternative medicine has not been associated with perceived psychological distress or poor compliance with standard treatment but with active coping behavior [18].

The use of TCM is related to culture and varying pattern have been reported globally and within countries in SSA [8, 9, 13, 14]. No research on the use of TCM among cancer patients in Lesotho has previously been done. Therefore, this study was conducted to determine the prevalence of TCM use, reason for use among cancer patients seen at the Senkatana oncology clinic, Maseru. The expected and actual benefits from TCM were also investigated. An investigation of whether patients informed their oncologist of their concurrent use of TCM and the reasons for disclosure or non-disclosure was further done.

## Methods

### The aim

The aim of the study was to assess the prevalence of the use of traditional and complementary medicines among cancer patients and to investigate the reasons for using such medicines.

### Study design

A cross-sectional, quantitative study was conducted at Senkatana Oncology clinic in May to June 2023. Cancer patients were interviewed using structured questionnaires about their use of complementary and alternative medicines.

### Study setting

Data was collected at the Senkatana oncology clinic. This clinic is located at the Botshabelo complex in Maseru. Senkatana center of excellence was established in 2004 to address the HIV/AIDS pandemic as the first site to pilot antiretroviral therapy (S + ART) in Lesotho. Senkatana oncology clinic was established through the Bristol Meyers Squibb Foundation (BMSF) Africa cancer disparities funding in 2020 as the first Lesotho cancer treatment center. Since its establishment, patients have been receiving treatment of solid cancers at the clinic and for haematologic cancers, patients are referred from the clinic to treatment centers in Bloemfontein, South Africa.

### Study population and sample determination

The study population is all cancer patients served by Senkatana oncology clinic including those who receive treatment at cancer centers in Bloemfontein, South Africa and are being followed up at the clinic. The study population was taken as 100 active patients seen monthly based on the files from the clinic. The sample size was calculated at 5% margin of error and 95% confidence interval using Slovin formula. The target sample size was set at 80. Convenience sampling was used for inclusion of patients in the study. Exclusion criteria encompassed cancer patients who could not communicate due to severe illness and pain, those with mental disorders and those who were not available at the time of data collection.

### Data collection instruments

A structured questionnaire administered was adopted from the validated questionnaire used in another African country; Nigeria [9]. The questionnaire was translated into Sesotho. It consisted of sociodemographic characteristics of the patients. Information captured in the questionnaire included their medical history; the cancer type and stage at diagnosis, the type of conventional treatment they have undertaken as well as the period patients have been on treatment. Data on patients traditional and complementary medicine use was taken; the type of TCM used, the reasons for use as well as whether they informed their healthcare practitioners of their use of TCM. Further data on the expected and actual benefit of TCM was gathered.

### Data gathering process

Data was collected by a pharmacy student who had been trained on data collection for a period of a week. A translated questionnaire was administered through an interview to patients queuing for cancer services at the Senkatana oncology clinic. Patients read and filled an informed consent form prior to an interview. Each interview took 90 min. Data collection was done twice a week and 5 patients were interviewed in a day. The data collection was done in a month and 1 week. Atleast ten (10) patients were interviewed every week for five weeks. A total of 50 patients participated in the study. This makes approximately 63% of the target calculated sample size.

### Data analysis

Data was captured into Microsoft excel® and analyzed using descriptive and inferential statistics. Data completeness was ensured prior to analysis. Results were summarized as count and percentages. Binary logistic regression was used to identify factors associated with satisfaction with the performance of TCM; independent variables studied were age and gender and the outcome variable being satisfaction with the performance of TCM.

## Results

### Demographic characteristics

All interviewed patients (*n* = 50, 100%) reported to be using TCM. As shown in Table 1, respondents consisted of 24 females (48%) and 26 males (52%) in the age range 14 to 82 years old. The median age was 45 years old and the majority of the study population was in the age group 35–44 years old. More than 60% of the study participants had high school education and beyond; being diploma, technical or university degree and 52% (*n* = 26) were married. Among participants who reported their employment status, 34% were unemployed while 25% were employed, 19% self-employed and 22% were retired. Majority of the study population (*n* = 47, 94%) were Christian and 25% (*n* = 12) were catholic.

**Table 1:** Demographic characteristics of study participants (*n* = 50)

	Female			Male		Total	
Age-group	Number	%		Number	%	Number	%
Under 18	0	0%		1	3.8%	1	2%
18–34	5	20.8%		5	19.2%	10	20%
35–44	7	29.2%		7	26.9%	14	28%
45–54	5	20.8%		3	11.6%	8	16%
55–64	5	20.8%		6	23.1%	11	22%
65+	2	8.4%		4	15.4%	6	12%
**Total**	**24**	100.0%		**26**	100.0%	**50**	100.0%
Median age (range)	*n* = 24	45 (26–82)		*n* = 28	45 (14–70)	*n* = 50	45 (14–82)
**Educational level**							
None	3	12.5%		1	3.8%	4	8%
Primary education	5	20.8%		9	34.6%	14	28%
Secondary/High school	7	29.2%		8	30.8%	15	30%
Technical/Diploma/University	9	37.5%		8	30.8%	17	34%
**Total**	**24**	100.0%		**26**	100.0%	**50**	100.0%
**Marital status**							
Married	11	45.8%		15	57.7%	26	52%
Not married	7	29.2%		7	26.9%	14	28%
Widow	5	20.8%		2	7.7%	7	14%
Divorced/Separated	1	4.2%		2	7.7%	3	6%
**Total**	**24**	100.0%		**26**	100.0%	**50**	100.0%
**Occupation**							
Employed	5	20.8%		3	11.5%	8	16%
Self-employed	2	8.3%		4	15.4%	6	12%
Unemployed	6	25%		5	19.2%	11	22%
Retired	3		12.5%	4	15.4%	7	14%
Missing	8		33.4%	10	38.5%	18	36%
**Total**	**24**		100.0%	**26**	100.0%	**50**	100.0%
**Religion**							
Traditional religion	0		0%	2	7.7%	2	4%
Catholic	8		33.3%	4	15.4%	12	24%
Pentecostal	2		8.3%	4	15.4%	10	20%
Anglican	1		4.2%	7	26.9%	8	16%
LEC	5		20.8%	2	7.7%	7	14%
Others	8		33.3%	6	23.1%	10	20%
Missing	0		0%	1	3.8%	1	2%
**Total**	**24**		100.0%	**26**	100.0%	**50**	100.0%

### Medical history and timespan of TCM use

**Fig. 1:**
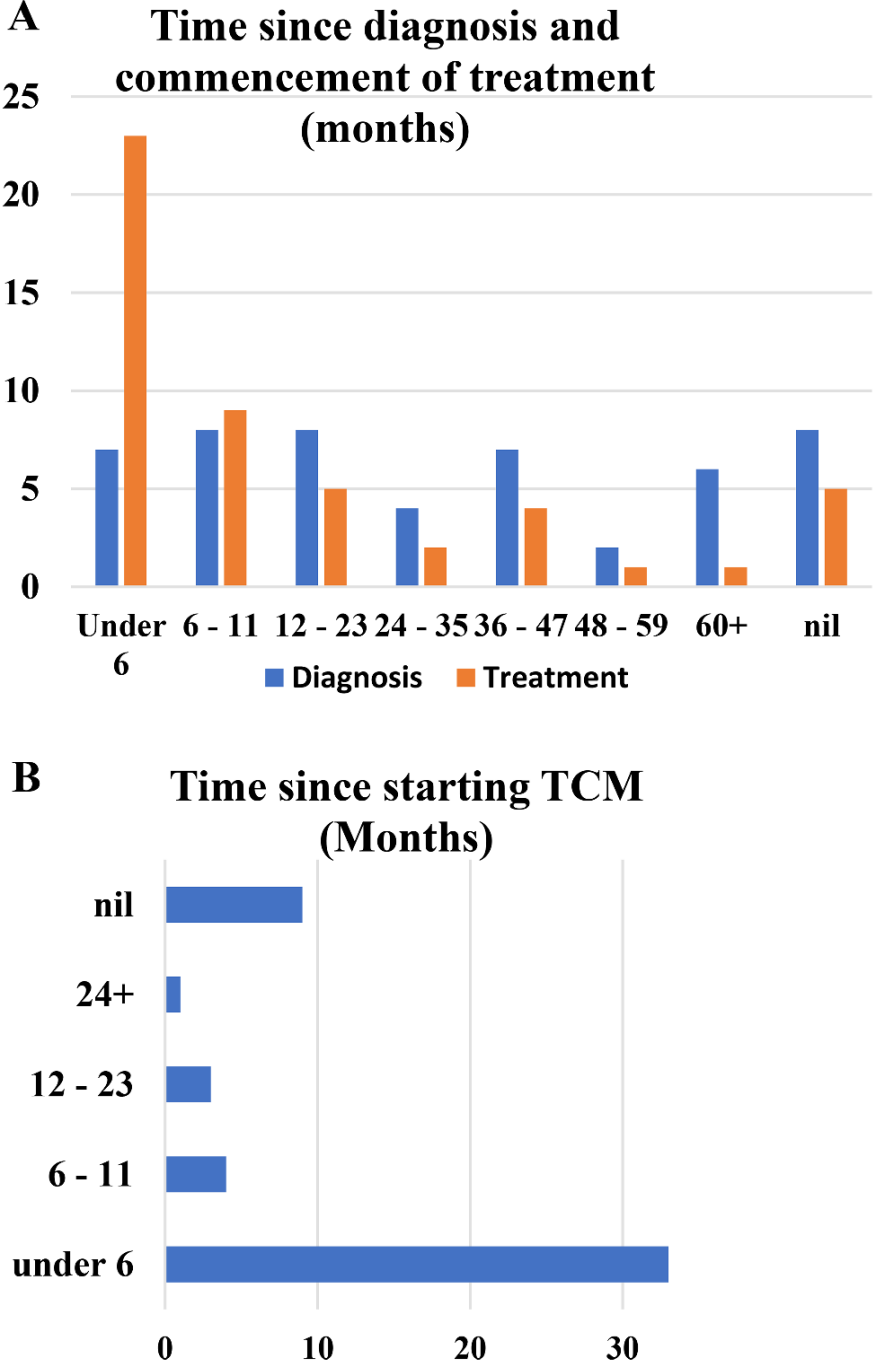
**A** Time (Months) since cancer diagnosis and commencement of treatment. **B** Time since starting TCM

The most prevalent cancer among participating males was prostate cancer and among females was cervical cancer as demonstrated in Table 2. Breast cancer prevalence was the same among females and male participants. A total of 7 patients (14%) consisted of haematological cancers and there were 2, 5 and 2 patients with bone, colon and ophthalmic cancer, respectively. One patient with Kaposi sarcoma was also interviewed. As demonstrated in Fig. 1, majority of the participants (*n* = 32, 64%) had been under treatment for less than a year. However, 54% of the participants had obtained their cancer diagnoses in the previous year or before then, up to more than 5 years ago. Majority of the study participants (*n* = 33, 66%) reported to have started using TCM less than 6 months ago. Conventional treatment modalities that interviewed patients had undergone included, chemotherapy, surgery and radiotherapy whereby 54% of the patients had undergone only chemotherapy and 38% had both surgery and chemotherapy.

**Table 2:** Cancer types among study participants (*n* = 50)

Cancer type	Female		Male		Total	
	Number	%	Number	%	Number	%
Blood	2	8.3%	5	19.2%	7	14%
Bone	1	4.2%	1	3.8%	2	4%
Breast	4	16.7%	4	15.4%	8	16%
Cervical	8	33.3%	0	0%	8	16%
Colon	2	8.3%	3	11.5%	5	10%
Esophageal	0	0%	1	3.9%	1	2%
Eye	2	8.3%	0	0%	2	4%
Kaposi sarcoma	0	0%	1	3.9%	1	2%
Ovary	4	16.7%	0	0%	4	8%
Prostate	0	0%	11	42.3%	11	22%
Vaginal	1	4.2%	0	0%	1	2%
**Total**	**24**	100.0%	**26**	100.0%	**50**	100.0%

### Type of TCM used, reasons for use and outcomes

Complementary medicines covered biological product, mind-body systems, alternative system, physical therapy and others. Biological products use was the most prominent with the highest average percentage usage (14.7%) as shown in Table 3. Among these products herbal drugs and special diet/ nutritional therapies & supplements both consisted of 42% usage among study participants. About 22% of patients reported that they practice faith/prayer healing and alternative systems had 10% usage among study participants while 12% of the participants reported to use massage as a physical therapy. An average of 2% study population had undergone other therapies including urine therapy, ritual sacrifice, scarification or animal sacrifice for therapy.

**Table 3:** Types of complementary medicines used by patients (*n* = 50)

Biological products	Frequency of use			Frequency of use	
	Number	%	Mind-body systems	Number	%
Herbal drugs	21	42%	Faith healing/Prayer house healing	11	22%
High dose/ mega vitamins	3	6%	Divination/ Incantations	4	8%
Forever Living Product	1	2%	Meditation	1	2%
Alo vera	5	10%	Visualization/ Vision	2	4%
GNLD product	1	2%	Hypnosis	1	2%
Nutri water	1	2%			
Medicinal tea	14	28%			
Green tea	11	22%	**Alternative systems**		
Kosagog tea	1	2%	Chinese medicine	5	10%
Special diet/ nutritional therapies & supplements	21	42%	Indian medicine	5	10%
Mineral Treatment	2	4%			
**Others**			**Physical therapy/body manipulations**		
Urine Therapy	1	2%	Osteopathy/ Bone setters	1	2%
Animal extracts	1	2%	Massage	**6**	12%
Local surgery/Scarification	1	2%	Manual healing (therapeutic Touch)	**3**	6%
Ritual Sacrifice	1	2%			

The most common reasons interviewed cancer patients gave for their use of complementary and alternative medicine were that complementary medicine is more in keeping with their beliefs and inner self (20%, *n* = 10) and that they want to take control of their own treatment and faith in their own hands (24%, *n* = 12). Table 4 demonstrates that the majority of patients (66%, *n* = 33) indicated that they just wanted to try everything that could help. Patients (*n* = 47, 94%) further reported that they had been using complementary medicine during the same period as they were using conventional treatment so that both may work to help each other.

**Table 4:** Reasons for deciding to use complementary medicine (*n* = 50)

	Number	%
Disappointed that conventional treatment is not working	5	10%
Conventional treatment is too toxic or too mutilating	5	10%
Complementary medicine is more in keeping with beliefs and inner self	10	20%
Want to take control of own treatment and faith in one’s own hands	12	24%
Conventional treatment is too mechanistic/technological and lacks human touch	1	2%
Just trying everything that can help	33	66%

It is demonstrated in Table 5 that study participants further reported that they expected complementary medicine to directly treat or cure the cancer (64%, *n* = 32), that it will boost the body’s ability to fight the cancer (34%, *n* = 17) and that it will relieve the symptoms of the cancer (22%, *n* = 11) Only one patient reported that they were expecting that the complementary medicine will relieve adverse effects of conventional treatment. Majority of the participants (*n* = 47, 94%) further reported that they had never abandoned conventional medicine for complementary medicine. The reasons that those who reported to had previously had to abandon conventional medicine were ‘*I felt healed and Facebook testimonies showed that this TCM has helped a lot of people’*, *‘I had bad pains in the stomach so when I took complementary medicine/herbs, pain was relieved a bit and I stopped vomiting while stopping conventional medicine’*, and ‘*I stopped because of their side effects’*.

**Table 5:** Expected benefits from using complementary medicine (*n* = 50)

	Number	%
It will directly treat/cure the cancer	32	64%
It will boost the body’s ability to fight the cancer	17	34%
It will allow to relax/sleep	5	10%
It will relieve adverse effects of conventional treatment	1	2%
It will relieve the symptoms of the cancer	11	22%
To do everything possible to fight the cancer	4	8%
It will improve the physical well being	4	8%
**Others** (‘*believe in herbal medicines’, ‘helps with stomach problems’*)	2	4%

**Fig. 2:**
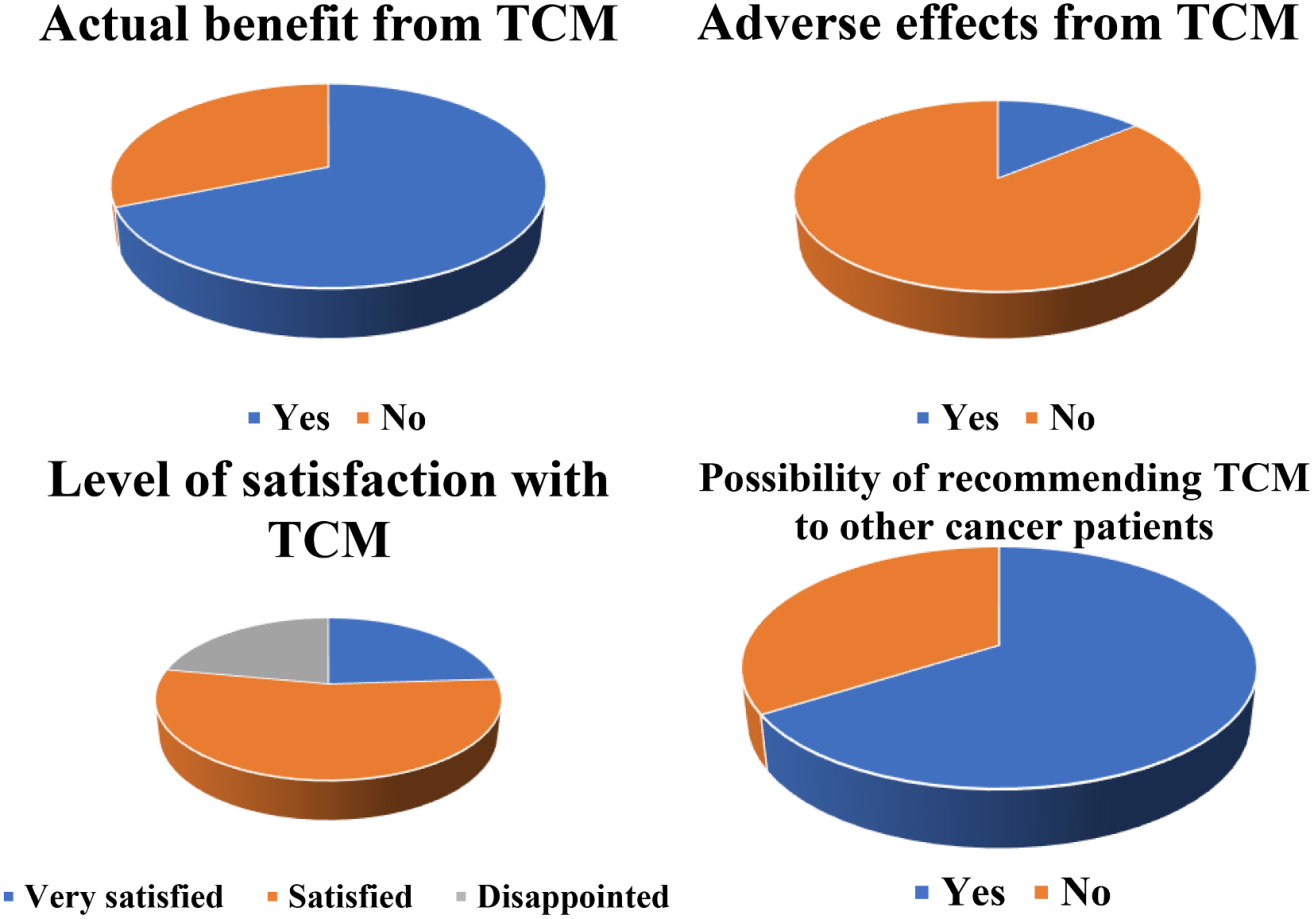
Reported benefit, adverse effects of TCM, level of satisfaction with TCM and the possibility of recommending it to other cancer patients

Majority of patients reported to have seen actual benefit from TCM as demonstrated in Fig. 2. Most of them (*n* = 27, 54%) were satisfied with their effects, and they did not experience any adverse effects (*n* = 47, 94%) from their use. Most patients (*n* = 33, 66%) admitted that they would recommend the use of TCM to other patients.

Cancer patients at the clinic (*n* = 48, 96%) reported that they did not inform their attending healthcare professionals of their use of TCM, and some of the reasons given for the non-disclosure included that the healthcare professionals have advised against the use of any other medicines besides what they are given at the clinic (*n* = 15, 65%), they did not find it necessary (*n* = 6, 26%) and they believe that the TCM used does not interact with chemotherapy treatment taken (*n* = 2, 8%). One cervical cancer patient mentioned that she did not inform the oncologist about this because the TCM was helping her and if she disclosed this, she was afraid that the oncologist will tell her to stop using TCM.

## Discussion

All interviewed patients (*n* = 50, 100%) reported to be using TCM. The median prevalence of use of TCM has been reported at 60% in Sub-Saharan Africa [8]. In South Africa, the prevalence of TCM use have been reported at 38.5% among the general population of Indians [9]. Respondents consisted of 24 females (48%) and 26 males (52%) in the age range 14 to 82 years old. The median age was 45 years old and the majority of the study population was in the age group 35–44 years old. This shows a pattern of cancer prevalence among young adults which is unlike several studies that have reported higher prevalence of cancer in the older population. More than 60% of the study participants had high school education and beyond; being diploma, technical or university degree and 52% (*n* = 26) were married. Reports have shown that the Lesotho literacy rate is at 81.02% in 2023 [19]. The most prevalent cancer among participating males was prostate cancer and among females was cervical cancer. This is consistent with the most common cancers in Lesotho [7]. Breast cancer prevalence was the same among females and male participants. Breast cancer incidence in Lesotho in the year 2020 was higher in females than males [7]. Globally, breast cancer has been related with the female gender due to its association with oestrogen [20].

The most common reasons interviewed cancer patients gave for their use of complementary and alternative medicine were that complementary medicine is more in keeping with their beliefs and inner self and that they want to take control of their own treatment and faith in their own hands. A scoping review done in the Sub-Saharan Africa showed that many cancer patients use TCM with the aim of getting rid of cancer symptoms, especially pain. They also hope to cure the cancer, improve physical and psychological well-being, treat toxicity of conventional cancer therapies as well as improve immunity [8]. The use of complementary and alternative medicine by cancer patients has not been associated with perceived distress or poor compliance with standard treatment but with active coping behaviour [18].

Majority of cancer patients who use TCM do not disclose this to their treating healthcare professionals and the disclosure have been reported to be as low as 32% in SSA [8]. The main reason for this non-disclosure being that cancer patients are advised to avoid using any other medicine besides what is prescribed at the healthcare facility.

## Conclusions

It is concluded that all interviewed cancer patients use TCM. The reasons for use among cancer patients keeping with their beliefs, inner self, taking control of their own treatment and faith in their own hands. The majority of patients indicated that they wanted to try everything that could help. The expected and actual benefits from TCM included directly treat or cure the cancer, boosting of the body’s ability to fight the cancer and relieve the symptoms of the cancer. Patients did not inform their oncologist of their concurrent use of TCM because they have been advised not to use other medicines besides what they are given at the clinic.

### Electronic supplementary material

Below is the link to the electronic supplementary material.

**Supplementary Material 1:** Questionnaire on the use of complementary medicine by cancer patients in Lesotho

## Data Availability

The datasets used and/or analysed during the current study are available from the corresponding author on reasonable request.

## Declarations

### Recommendations

Further studies on the outcomes of cancer patients who use TCM relative to those who do not, need to be conducted. The pre-clinical effects of the most commonly used traditional and complementary medicines in Lesotho need to be studied as well.
