# Supplementary material for: A preliminary, quantitative study on the use of traditional and complementary medicine by cancer patients seen at the Senkatana oncology clinic, Maseru, Lesotho
Source: BMC Complement Med Ther. 2024 Apr 1;24:136. doi: 10.1186/s12906-024-04388-3 (PMC10983638; doi:10.1186/s12906-024-04388-3)
Supplement: Supplementary file 1 — Supplementary Material 1: Questionnaire on the use of complementary medicine by cancer patients in Lesotho [file 12906_2024_4388_MOESM1_ESM.pdf]

ANNEXURE

QUESTIONNAIRE ON THE USE OF COMPLEMENTARY MEDICINE BY CANCER PATIENTS IN LESOTHO

This questionnaire is designed to find out to what degree cancer patients in our clinic employ complementary remedies in the treatment of their cancer. It is only a research work. The answers you give to the questions here will in no way influence how we are going to treat you as an individual. The findings of this research will help us to understand our cancer patients better and therefore help us to improve the care that we render to them. We implore you to give us as honest an answer to each question as possible. You may choose not to respond to any of the questions you are not comfortable with. We guarantee you confidentiality.

CASE NOTE NO:____________________

DEMOGRAPHIC DATA

1. Age: --------------

2. Sex: Male-------- Female---------

3. Marital status: (A) Married (B) Not married (C) Widow (D) Divorced/Separated

4. Highest level of education attained:
(A) Non (B) Primary (C) Post primary/Secondary
(D) University/Polytechnic/College of education

5. Profession/Occupation: ---

6. Level of Income per month:
(A) Less than M1,000.00/month (B) Less than M5,000.00/month
(C) Less than M10, 000.00/month (D) More than M10,000.00/Month

7. Religion: (A) Traditional religion (B) Catholic (C) Pentecostal
(D) Anglican (E) LEC (F) Others (specify) --

8. If you are to rate your self, how religious would you say you are?

(A) Extremely religious

(B) Very religious

(C) Religious

(D) Not really religious

(E) No religious ideas

CONVENTIONAL CANCER TREATMENT

9. Type of cancer? ---(confirm from case note)

10. Stage of cancer? ---(confirm from case note)

11. How long have you had this disease? --

12. How long have you been on treatment for this disease? ----------------------------------

13. What types of treatment have you received in the past for this cancer?

(A) Drugs for cancer

(B) Surgery (specify)

(C) Radiotherapy

(D) Others (specify)

14. What type of treatment are you currently receiving for this disease? ---------------------
----

COMPLEMENTARY MEDICINE USE

15. Have you used anything other than that given to you by a medical doctor to treat this cancer?

(A) Yes (B) No

16. Below is a list of complementary medicine people have used to treat cancer. We will like to know which ones you have used in the past, which ones you have used since this cancer started and which ones you think you may use in future. You should mark all that applies to you for each medicine

<u>Biological Products:</u>

	**Used in the past**	**Used since this cancer**	**Hope to use**
Herbal drugs	-------	-------	-------
High dose/ mega vitamins	-------	-------	-------
Forever Living Product	-------	-------	-------
Tuja 1000	-------	-------	-------
Alo vera	-------	-------	--------
GNLD product	------	-------	-------
Nutri water	------	------	------
Medicinal tea	-------	-------	-------
Green tea	--------	--------	--------
Kosagog tea	--------	--------	--------
Special diet/ nutritional therapies & supplements	-------	-------	-------
Mineral Treatment	-------	-------	-------

SPIRITUAL THERAPY/MIND-BODY SYSTEMS

	Used in the past	Used since this cancer	Hope to use
Faith healing/ prayer house healing	-------	-------	-------
Divination/ Incantations	-------	-------	-------
Meditation	-------	-------	-------
Visualization/ Vision	-------	-------	-------
Hypnosis	-------	-------	-------
Psychic Therapy	-------	-------	-------
Mind-body Technique	-------	-------	-------
Mental Imagery	-------	-------	-------

ALTERNATIVE SYSTEMS

	Used in the past	Used since this cancer	Hope to use
Chinese Medicine	-------	-------	-------
Indian Medicine	-------	-------	-------
Acupuncture	-------	-------	-------
Homeopathy	-------	-------	-------

PHYSICAL THERAPY/BODY MANIPULATIONS

	**Used in the past**	**Used since this cancer**	**Hope to use**
Chiropractic	-------	-------	-------
Osteopathy/ Bone setters	-------	-------	-------
Massage	-------	-------	-------
Manual healing (therapeutic Touch)	-------	-------	-------

ENERGY THERAPIES

Bioelectro-magnetics	-------	-------	-------
Oxygen/ozone Treatment	-------	-------	-------

OTHERS

Blood letting Couping	-------	-------	-------
Local surgery/ Scarification	-------	-------	-------
Ritual Sacrifice	-------	-------	-------
Urine therapy	-------	-------	-------
Folk remedies (specify)	-------	-------	-------
Black stone	-------	-------	-------
Shark cartilage	-------	-------	-------
Python fat	-------	-------	-------
Animal extracts	------	-------	-------

17. Are there other types of complementary medicine not listed above which you have used?
    (A) Yes (specify) --- (B) No-----

18. If you have used any complementary medicine in this cancer, for how long have you used it? -------------

19. How frequently have you been using complementary medicine in this cancer?
    (A) Daily (B) weekly
    (C) Occasionally (D) Only once

20. Which of the following sentences explains how you have been using the complementary medicine and conventional treatment?

    (A). You started conventional treatment only when you stopped complementary medicine

    (B) You started complementary medicine only when you finished conventional treatment

    (C) You were using complementary medicine during the same period as you were using conventional treatment so that both will work to help each other

21. How do you hope to use your complementary medicine and the orthodox treatment as your treatment progresses?
    (A) Use the two to help each other
    (B) Use the complementary medicine instead of the orthodox medicine
    (C) Use orthodox medicine instead of complementary medicine

22. How frequently have you visited a complementary medicine practitioner since you were diagnosed to have cancer? (A) Non (B) Once (D) Several times

23. What are your reasons for deciding to use complementary medicine (you can choose more than one) (A). You were disappointed that conventional treatment is not working

(B). Conventional treatment is too toxic or too mutilating

(C). You think complementary medicine is more in keeping with your beliefs and your inner self

(D). you want to take control of your treatment and your faith in your own hands

(E). Conventional treatment is too mechanistic/technological and lacks human touch

(F) You are just trying every thing that can help

(G) Others (specify plse)

24. What benefits were you hoping to get from the complementary medicine you used in this cancer? (you can choose more than one)

(A) It will directly treat/cure your cancer

(B) It will boost your body's ability to fight the cancer

(C) It will allow you to relax/sleep

(D) It will clean up your wounds

(E) It will relieve symptoms of conventional treatment which you are receiving

(F) It will relieve the symptoms of the cancer

(G) It will improve your psychological/emotional well being (hope, optimism)

(H) to do every thing possible to fight the cancer

(I) It will improve your physical well being

(J) Others (specify)

25. Have you obtained any particular benefit from the complementary medicine you used?

(A) Yes (specify) ---

(B) No

26. Did you experience any unwanted effect from the complementary medicine you used in this cancer?

(A) Yes (specify) --

(B) No

27. How satisfied are you with the performance of the complementary medicine you used in this cancer?

(A) Very satisfied (B) Satisfied (C) Disappointed

28. Would you use complementary medicine again for cancer or recommend it for someone with cancer?

(A) Yes (B) No

29. Have you had to abandon conventional treatment for complementary medicine since you started using complementary medicine?.

    (A) Yes (B) NO

30. What were your reasons for abandoning orthodox treatment for complementary medicine since you started complementary medicine?---
    ----
    ----

31. Did you mention to the doctor in charge of this your cancer management that you have used/are using complementary medicine? (A) Yes (B) No

32. If your doctor is not aware that you have used/are using complementary medicine, what is it that makes you feel unwilling to discuss it with him/her?---
    ----

    ----

33. How did you come to know of the complementary medicine you are using/have used (you can choose more than one)

    (A) From health personnel outside of hospital setting

    (B) From health personnel in the hospital

    (C) From Friends

    (D) From family members

    (E) From complementary medicine practitioner

    (F) From Mass media (TV, newspaper, radio, magazines)

    (G) From your church/religious group

    (H) From Migrant advertisers

    (I) From other patients

    (J) Others (specify) --

34. How do you get your supply of complementary medicine?

  (A) From friends

  (B) From relations

  (C) From complementary medicine practitioner

  (D) You buy from the market

  (E) From your church

  (F) Others (specify) --

35. How much do you estimate that you have spent on complementary medicine in the last one year? ---------
----

36. What particular aspects of your complementary medicine would you have wished is available in orthodox medicine?--
----
----

37. Does any body supervise/guide you in the complementary medicine you use?

  (A) Yes (B) No
